# Recurrence of Adult Granulosa Cell Tumors: The Factors Affecting Secondary Recurrence and Survival After Recurrence

**DOI:** 10.7150/jca.127596

**Published:** 2026-03-17

**Authors:** Hasan Volkan Ege, Derman Başaran, Murat Gültekin, Nejat Özgül, Nurattin Boran, Sevgi Koç, Yaprak Üstün, Caner Çakır, Dilek Yüksel, Okan Oktar, Gökçen Ege, Abdurrahman Alp Tokalıoğlu, Mustafa Şahin, Yeşim Uçar, Fatih Kılıç, Okan Aytekin, Burak Ersak, Mehmet Ünsal, Özlem Moraloğlu Tekin, Çiğdem Kılıç, Özgür Koçak, Çağatayhan Öztürk, Salih Taşkın, Fırat Ortaç, Işın Üreyen, Tayfun Toptaş, Sevda Baş, Mehmet Ali Narin, Tolga Taşçı, Gökhan Uçar, Mehmet Ali Şendur, Burak Civelek, Doğan Uncu, Özgür Erdoğan, Muzaffer Sancı, Hakan Raşit Yalçın, İlker Selçuk, Taner Turan

**Affiliations:** 1 Division of Gynecologic Oncology, Department of Obstetrics and Gynecology, Faculty of Medicine, Hacettepe University, Ankara, 06800, Türkiye.; 2 Department of Gynecologic Oncology, Etlik Zubeyde Hanim Women's Health Training and Research Hospital, Faculty of Medicine, University of Health Sciences, Ankara, 06010, Türkiye.; 3 Department of Gynecologic Oncology, Ankara Bilkent City Hospital, Faculty of Medicine, University of Health Sciences, Ankara, 06800, Türkiye.; 4 Department of Gynecology and Obstetrics, Corum Hitit University Medical School, Corum, 19040, Türkiye.; 5 Department of Gynecology and Obstetrics, Ankara University Medical School, Ankara, 06100, Türkiye.; 6 Department of Gynecological Oncology, Antalya Training and Research Hospital, Health Science University, Antalya, 07100, Türkiye.; 7 Department of Gynecologic Oncology, Adana City Training and Research Hospital, Adana, 01230, Türkiye.; 8 Department of Gynecological Oncology, Bahcesehir University Medical School, Istanbul, 34349, Türkiye.; 9 Department of Medical Oncology, Ankara Bilkent City Hospital, Ankara, 06800, Türkiye.; 10 Department of Gynecologic Oncology, Tepecik Training and Research Hospital, University of Health Sciences, Izmir, 35020, Türkiye.; 11 Zekai Tahir Burak Women's Health Training and Research Hospital, Department of Gynecologic Oncology, Faculty of Medicine, University of Health Sciences, Ankara, 06230, Türkiye.

**Keywords:** adult granulosa cell tumors, complete cytoreduction, recurrence, salvage chemotherapy

## Abstract

**Introduction:**

Adult-type granulosa cell tumors (AGCTs) are rare ovarian neoplasms with a low overall incidence of recurrence, and also data on secondary recurrence and survival after relapse remain limited. This study aimed to identify factors associated with secondary recurrence and survival after recurrence in patients with recurrent AGCTs.

**Methods:**

This multicenter retrospective study included 52 patients with recurrent AGCTs identified among 484 patients treated between 2000 and 2023. Clinical characteristics, treatment modalities, and outcomes were analyzed, with a particular focus on factors associated with secondary recurrence and survival after first recurrence. Recurrence-free survival and overall survival after first recurrence (OS-FR) were evaluated using Kaplan-Meier analysis.

**Results:**

The mean follow-up duration was 99.2 ± 61.5 months. Secondary recurrence occurred in 17 patients (32.7%). A serum CA-125 level >35 U/mL at the time of first recurrence was significantly associated with an increased risk of secondary recurrence (p=0.01). Factors significantly associated with improved OS-FR included a CA-125 level ≤35 U/mL at initial diagnosis and at first recurrence, absence of residual disease following surgery for the first recurrence, and administration of salvage chemotherapy (all p<0.05). In subgroup analysis, salvage chemotherapy was associated with improved OS-FR in patients with residual disease or those who did not undergo surgery (p < 0.01), but not in patients who achieved complete cytoreduction (p = 0.67).

**Conclusions:**

Secondary recurrence remains a significant clinical challenge in AGCTs. Serum CA-125 levels, surgical outcomes at first recurrence, and the use of salvage chemotherapy may help management strategies in recurrent disease.

## Introduction

Granulosa cell tumors (GCTs) account for approximately 5% of all ovarian cancers and 70% of sex cord-stromal tumors 1, 2. Due to slow progression and frequent diagnosis at an early stage, GCTs have a favorable prognosis compared to epithelial ovarian tumors 3, 4. Based on histopathological features, GCTs are classified into two subtypes: Adult-type and juvenile-type 5. Adult-type granulosa cell tumors (AGCTs) typically occur in perimenopausal and postmenopausal women similar to epithelial ovarian cancers; surgery remains the cornerstone of management 1. The objective of surgery should be to achieve complete cytoreduction. Although adjuvant chemotherapy is included in the treatment algorithms of several guidelines 1, 6, its efficacy remains controversial, with conflicting results reported 3, 5, 7. Adjuvant chemotherapy is used in Stage II-IV disease. Adjuvant therapy could be given in risky cases such as tumor rupture or Stage IC disease 1.

Despite their favorable prognosis, AGCTs have a recurrence rate of 10-64% 1, 5, 8-10. Recurrences are usually characterized by slow growth and often occur at multiple sites 1. Given the rarity of AGCTs, standardized treatment approaches for recurrent disease are lacking 2, 4, 10. The knowledge on the management of recurrent AGCT is limited to a small number of retrospective series and case reports 3.

This multicenter study aimed to evaluate clinical characteristics, treatment strategies, and outcomes in patients with recurrent AGCTs. Specifically, the primary objective was to identify prognostic factors associated with secondary recurrence. Additionally, parameters influencing postrecurrence survival were analyzed.

## Materials and Methods

**Figure 1:**
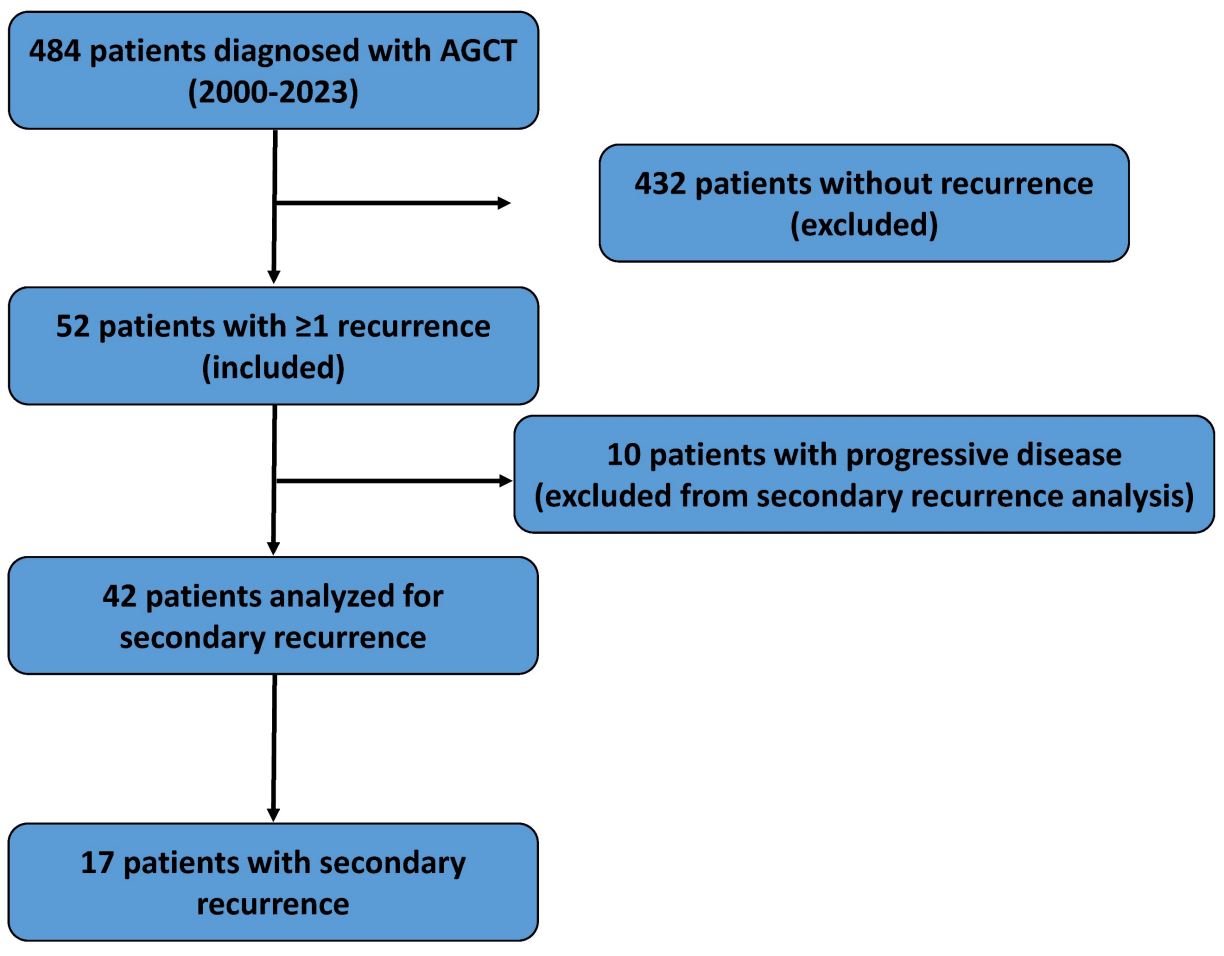
Flowchart illustrating patient selection, inclusion and exclusion criteria, and the final study population included in the analyses.

We retrospectively reviewed medical records of 484 patients diagnosed with AGCT across 10 gynecologic oncology centers between January 2000 and December 2023. Of these, 52 patients (10.7%) experienced at least one recurrence. A flowchart summarizing patient selection and study design is provided in Figure 1.

Demographic and clinical data were extracted from institutional databases. No direct patient contact, including telephone interviews, was performed for data collection. Patients were staged according to the 2014 International Federation of Gynecology and Obstetrics (FIGO) classification of ovarian tumors.

Recurrence was diagnosed based on radiological imaging demonstrating new or progressive lesions compatible with AGCT recurrence during follow-up, supported by clinical or operation findings, and, when available, histopathological confirmation. Recurrences were classified as pelvic, abdominal, or extra-abdominal. This classification was based on the 2014 FIGO criteria. Presence of malignant ascites at recurrence was classified as abdominal recurrence. Rupture was defined as preoperative spontaneous rupture or intraoperative capsule rupture documented in operative or pathology reports.

Treatment outcomes were evaluated according to the RECIST criteria 11. Patients were categorized into one of four groups: complete response (CR), partial response (PR), stable disease (SD), and progressive disease (PD). Patients with PD were excluded from further recurrence analyses.

Recurrence-free survival (RFS) was defined as the interval from initial diagnosis to the first recurrence. Overall survival after first recurrence (OS-FR) was defined as the time from the diagnosis of the first recurrence to death or last follow-up. Secondary recurrence was analyzed as a categorical outcome (presence or absence). Owing to not available data, survival analyses beyond the after-secondary recurrence were not performed.

Statistical analyses were conducted using SPSS version 23 (IBM Corp., Armonk, NY, USA). Categorical variables were summarized as numbers and percentages, and compared between groups using the chi-square test. Normality of continuous variables was assessed using the Kolmogorov-Smirnov tests. Continuous variables with normal distribution were expressed as mean ± standard deviation (min-max), whereas non-normally distributed variables were expressed as median (interquartile range [IQR]) (min-max). Group means were compared using the independent-samples Student's t-test or Mann-Whitney U test, as appropriate. Survival analyses were performed using the Kaplan-Meier method, and differences between survival curves were assessed with the log-rank test. A p-value < 0.05 was considered statistically significant.

## Results

The median follow-up duration of the entire AGCT cohort was 89 months (IQR: 60.8-123.8). The clinical features of the 52 included patients are summarized in Table 1. The mean age at diagnosis was 53.4 years (range, 33-82). Pelvic pain was the most frequent presenting symptom (30.8%), and the majority of patients (86.5%) underwent hysterectomy at the time of initial diagnosis. Pelvic lymph node dissection was performed in 37 patients (71.2%), with lymph node metastasis identified in eight cases. Among the 52 patients who had experienced at least one recurrence, 48.1% (n=25) had stage I disease at the time of initial diagnosis. Adjuvant chemotherapy was administered to 55.8% of patients (n=29), most commonly BEP (bleomycin plus etoposide plus cisplatin). The rate of adjuvant chemotherapy was 40% in stage I and 80% in stage II or higher. Two patients also received radiotherapy.

Median RFS was 55.2 months (range, 3-240), and median OS-FR was 35.0 months (range, 3-210). The first recurrence was predominantly multifocal (55.8%) (Table 2). Recurrences were most frequently observed in the pelvis (73.1%), while isolated extra-abdominal recurrence occurred in four patients, including two pulmonary, one inguinal, and one abdominal skin recurrence. At first recurrence, 84.6% of patients (n=44) underwent surgical treatment, with complete cytoreduction achieved in 90.9% (n=40) of these cases. Salvage chemotherapy was administered in 73.1% (n=38). A complete response was achieved in 48.1% (n=26), whereas progressive disease developed in 22.7% (n=10).

Secondary recurrence occurred in 17 patients (32.7%). Of these, 12 underwent surgery and seven of them also received salvage chemotherapy. Five patients who did not undergo surgery received chemotherapy alone. Overall, 58.9% of patients achieved CR or PR (n=10). Excluding cases with PD, tertiary recurrence occurred in five patients, and a fourth recurrence was observed in one patient at 82 months, which was again managed surgically (Table 2).

Demographic and clinical characteristics of the patients were analyzed to identify factors associated with secondary recurrence. The analysis included 42 patients who did not have PD after the first recurrence. A total of 22 parameters related to patient characteristics, disease features at initial diagnosis and first recurrence, and administered treatments were evaluated (Table 3). A CA-125 level >35 U/mL at first recurrence was the only factor significantly associated with an increased risk of secondary recurrence (4/10 vs. 0/16, p = 0.01).

**Figure 2:**
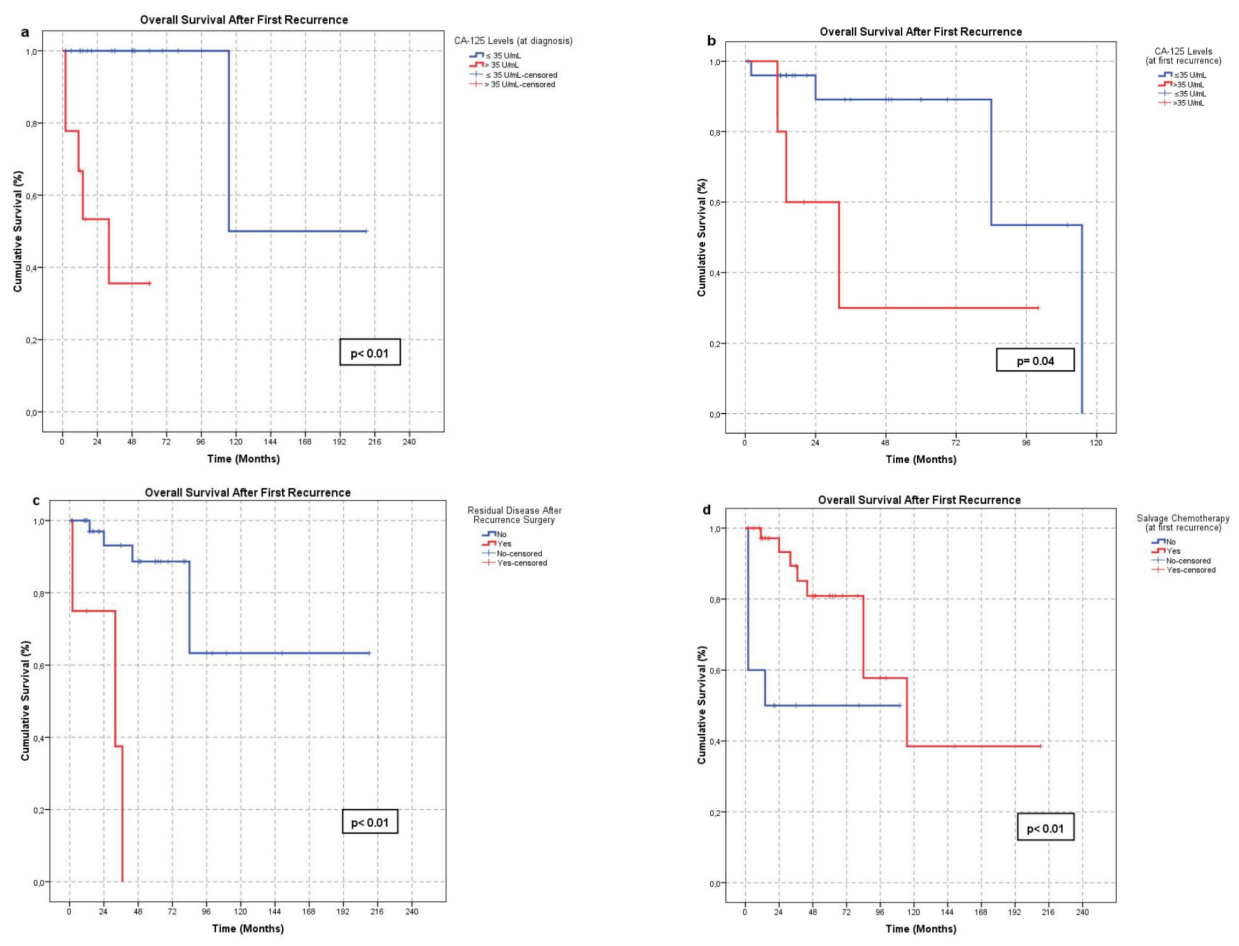
Factors significantly associated with OS-FR: **a.** Serum CA-125 level ≤35 U/mL at initial diagnosis, **b.** Serum CA-125 level ≤35 U/mL at first recurrence, **c.** Absence of residual tumor following surgery at first recurrence, **d.** Administration of salvage chemotherapy at first recurrence

**Figure 3:**
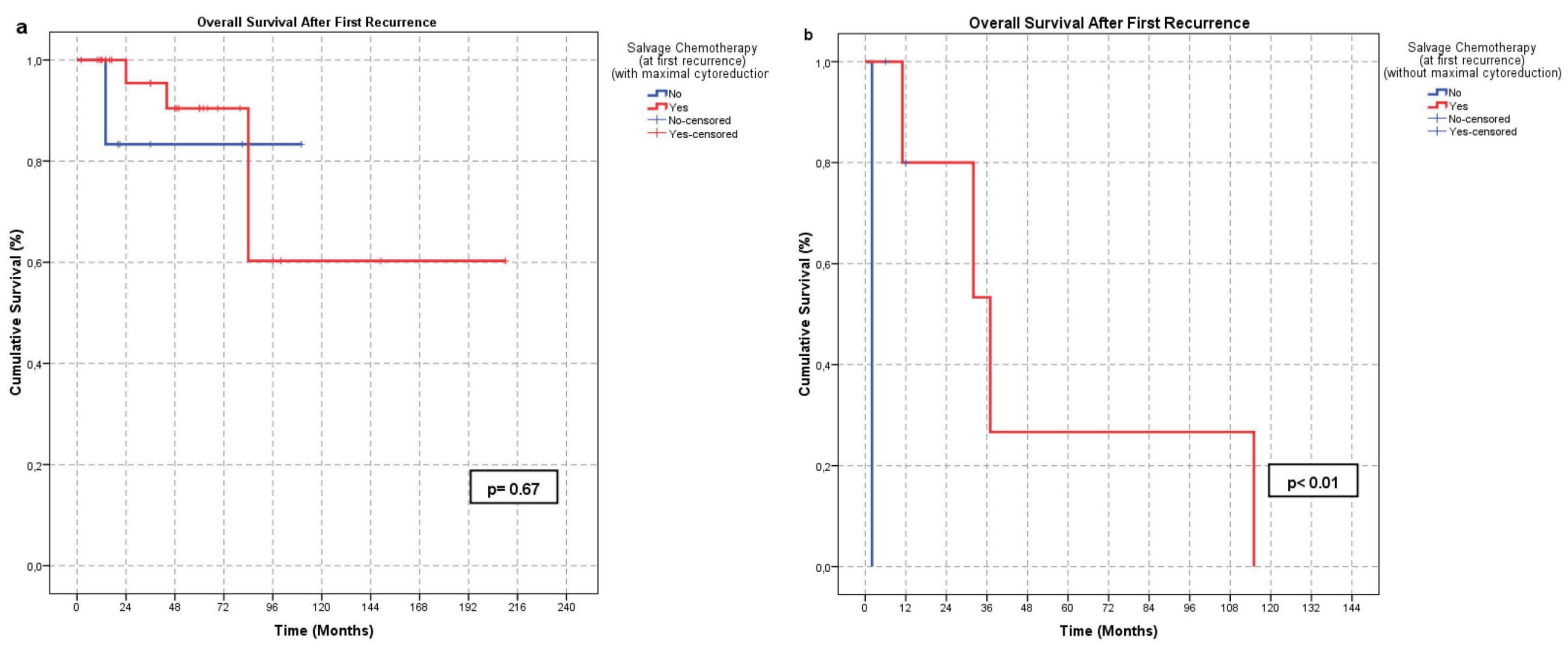
Effect of salvage chemotherapy on OS-FR according to surgical outcomes in the first recurrence: **a**. no significant difference in the group that undergone complete cytoreduction at first recurrence (p < 0.01) **b**. Significant effect in the group in which complete cytoreduction or surgical treatment was not performed (p = 0.67).

Factors affecting OS-FR were also evaluated. Fifteen parameters were analyzed (Table 4). A CA-125 level ≤35 U/mL at initial diagnosis (21/31) (p < 0.01) and at first recurrence (22/26) (p = 0.04), absence of residual disease after surgery at first recurrence (40/52) (p < 0.01), and administration of salvage chemotherapy following first recurrence (38/48) (p < 0.01) were significantly associated with improved OS-FR (Figure 2). In subgroup analysis, the survival benefit of salvage chemotherapy was evident in patients who did not undergo surgery or had residual disease following surgery (p < 0.01), whereas no significant benefit was observed in patients without residual tumor (p = 0.67) (Figure 3). No other parameters were significantly associated with OS-FR. Among patients with secondary recurrence, 10 achieved a treatment response (CR or PR). Due to the limited number of cases, further analysis of factors affecting OS-FR after secondary recurrence was not feasible.

## Discussion

AGCT is a rare malignant neoplasm classified within the sex cord-stromal tumor group. Different recurrence rates have been reported 3, 8-10. The disease usually progresses slowly and recurrence may develop years later 2-4, 10, 12. Due to its rarity and the tendency for late recurrence, there is no consensus on the optimal management of recurrent disease 8, 13. The few studies conducted on this topic provide information on a limited number of cases 3, 10, 14. Given the limited data on factors predicting secondary recurrence in AGCT, this study primarily aimed to identify clinical and treatment-related parameters associated with the development of secondary recurrence following the first relapse.

In our study, 52 patients diagnosed with AGCT who experienced at least one recurrence were analyzed. Our aim was to identify clinical and pathological factors that may increase the risk of a secondary recurrence and influence OS-FR. The identification of specific risk factors associated with secondary recurrence highlights the potential contribution of our study to the current literature. A CA-125 level >35 U/mL at the time of first recurrence was the only factor significantly associated with an increased risk of secondary recurrence. Furthermore, a CA-125 level ≤35 U/mL at both the time of initial diagnosis and first recurrence, absence of residual tumor following surgery for the first recurrence, and administration of salvage chemotherapy at the time of first recurrence were significantly associated with improved OS-FR. Additionally, in the group that underwent complete cytoreduction at first recurrence, salvage chemotherapy did not have a significant impact on OS-FR.

Even when diagnosed at an early stage, AGCT still carries a risk of recurrence, including in disease confined to a one ovary 14. The recurrences most frequently occur in the pelvic region and often present as multiple foci 3, 10, 14. Surgical treatment is the most commonly chosen approach for managing recurrence. However, the use of chemotherapy varies significantly between studies 3, 14.

In our series, 48.1% of patients were diagnosed with stage I disease. The first recurrence occurred in the pelvic region in 73.1% of cases, of which 55.8% were multifocal. Isolated extra-abdominal recurrence was observed in four patients (two in the lungs, one in the abdominal skin and one in the inguinal region). Of the patients who underwent surgery at the time of the first recurrence, complete tumor resection was achieved in 90.9%. Additionally, 73.1% of patients received salvage chemotherapy following the first recurrence.

Although AGCT is a rare tumor, several studies have investigated factors associated with recurrence risk. In our study, we evaluated 22 parameters potentially related to secondary recurrence. We found that a serum CA-125 level >35 U/mL at the time of the first recurrence was significantly associated with an increased risk of secondary recurrence. All patients with a CA-125 level above 35 U/mL developed secondary recurrence, compared to a rate of 27.3% among those with a level below 35 U/mL. Similarly, Huang *et al.* reported that elevated CA-125 levels at diagnosis were associated with an increased risk of recurrence 15. However, the strength of this finding in our study is limited by the small number of patients and the fact that CA-125 is not routinely used for AGCT follow-up. The use of markers such as anti-mullerian hormone, inhibin, and estradiol is recommended for the diagnosis and follow-up of AGCT 13.

Mangali *et al.* reported that the presence of residual disease following surgery at the initial diagnosis was associated with an increased risk of recurrence or progression (p=0.04). However, this effect was not observed following secondary cytoreductive surgery (p=0.12) 3. Similarly, Gu *et al.* found that the absence of residual lesions after surgery significantly reduced the frequency of recurrence (p<0.01) 10. In our cohort, the presence of residual disease after surgery did not significantly affect the risk of secondary recurrence (p=0.56). This high rate of complete cytoreduction may have limited the ability to detect a potential positive impact on the risk of secondary recurrence.

AGCT recurrences typically occur in the long term 2-4. There is conflicting evidence of the prognostic impact of early recurrences. While Gu *et al.* reported that a progression-free survival (PFS) >60 months did not affect recurrence rates, Zhao *et al.* identified PFS ≥61 months as an independent risk factor for repeated recurrence in their study, in which the secondary recurrence rate was 60% 10, 14. In our study, there was no significant difference in the risk of secondary recurrence between patients with a first recurrence occurring within 60 months and those with a first recurrence occurring beyond this period (p=0.12).

The impact of adjuvant therapy on risk of secondary recurrence remains controversial. Mangali *et al.* found no significant difference in secondary recurrence rates between patients who underwent surgery alone and those who received surgery plus chemotherapy at first recurrence (p=0.86) 3. Similarly, Gu *et al.* reported that adjuvant chemotherapy did not significantly affect recurrence rates (p=0.06) 10. In contrast, Zhao *et al.* observed that patients treated with surgery or chemotherapy alone had a higher risk of secondary recurrence compared to those who received combined surgery and chemotherapy 14. In our study, administration of chemotherapy either at the time of initial diagnosis or at first recurrence was not significantly associated with the risk of secondary recurrence.

Recurrences of AGCT frequently occur in the pelvic region and tend to be multifocal 10, 14. In our study, recurrences were most commonly observed in the pelvic region (73.1%) and were predominantly multifocal (55.8%). On univariable analysis, neither multifocality nor location at first recurrence significantly affected the risk of secondary recurrence. These findings are consistent with previous studies 10, 14. The high rate of complete cytoreduction achieved in these patients may have minimized the effect that the number and location of recurrences have on the risk of subsequent recurrence.

In addition to evaluating secondary recurrence, we also investigated factors influencing OS-FR, as a clinically meaningful secondary outcome. Thirteen parameters that could potentially influence survival after the first recurrence were evaluated. The CA-125 level of ≤35 U/mL both at initial diagnosis and at first recurrence, absence of residual tumor after surgery for the first recurrence, and administration of salvage chemotherapy in the first recurrence were all significantly associated with prolonged OS-FR. To our knowledge, there are no studies in the literature directly comparing CA-125 levels and their potential impact on survival in AGCT patients.

Surgery is recommended in the management of AGCT, both at initial diagnosis and in recurrent disease 1, 5, 6. The aim of surgical treatment should be complete cytoreduction. The presence of residual tumor after surgery was associated with poor prognosis. Seagle *et al.* found that residual disease was independently associated with an increased hazard of death 7. The beneficial impact of cytoreductive surgery on survival in recurrent disease was also demonstrated in a study by How *et al.*, where cytoreductive surgery was associated with a significant improvement in both progression-free survival and overall survival 16. Zhao *et al.* similarly showed that cytoreductive surgery had a significant positive impact on postrecurrence survival (p<0.01) 14. In contrast, Mangini *et al.* reported that while complete surgical staging significantly affected RFS, it did not have a similar effect on overall survival 3. In our study, complete cytoreduction at first recurrence was significantly associated with prolonged OS-FR. These results underline the importance of surgical efficacy in cases of recurrent AGCT and emphasize the value of maximal surgical effort in managing recurrent disease.

The role of adjuvant chemotherapy remains controversial 6. In a large cohort study involving 2680 patients, Seagle *et al.* reported that adjuvant chemotherapy did not significantly affect overall survival 7. Park *et al.* analyzed the efficiency of chemotherapy in relation to disease stage. Although chemotherapy had no significant impact on 5-year disease-free survival in patients with early-stage disease, it significantly improved disease-free survival in those with stage III-IV disease 17. In recurrent disease, Zhao *et al.* demonstrated that the combination of chemotherapy and surgery significantly improved recurrence-progression-free survival compared to surgery or chemotherapy alone 14. However, Gu *et al.* reported that chemotherapy administered at recurrence (given to 67.1% of patients) had no significant effect on either post-recurrence overall survival or post-recurrence progression-free survival 10. In our series, patients who received chemotherapy at the time of first recurrence had significantly longer post-recurrence overall survival. This effect was particularly evident in patients who did not undergo surgery or had residual disease following surgery. In contrast, chemotherapy did not confer an additional survival benefit in patients with no residual disease after surgery. Our findings support the administration of chemotherapy at first recurrence, particularly in patients who are not suitable candidates for surgery or in those with residual tumor after surgical treatment.

In the diagnosis and follow-up of AGCT, anti-mullerian hormone, inhibin, and estrogen are commonly used biomarkers 13. CA-125 is primarily used in the diagnosis and monitoring of epithelial ovarian cancers. In our study, we investigated the association between CA-125 levels and OS-FR. It was demonstrated that CA-125 levels ≤35 U/mL, both at initial diagnosis and at first recurrence, were significantly associated with prolonged OS-FR.

The retrospective design and lack of molecular data are notable limitations. Furthermore, biomarker data such as anti-mullerian hormone and inhibin were not uniformly available. Due to the limited number of patients experiencing secondary recurrence, survival analyses beyond the first recurrence could not be performed. Despite these limitations, the multicenter design and relatively large sample size strengthen the validity of our findings. In this regard, our study makes a meaningful contribution to the literature.

## Conclusion

Adult-type granulosa cell tumors are rare neoplasms characterized by late recurrences and a tendency towards multiple relapses. Serum CA-125 levels may be predictive of the risk of secondary recurrence and survival following the first recurrence. Complete cytoreduction in the event of recurrence is associated with prolonged survival and should be the primary surgical management goal. For patients for whom complete cytoreduction cannot be achieved, or for whom surgery is not feasible at the time of the first recurrence, salvage chemotherapy may offer a survival benefit.

## Figures and Tables

**Table 1:** Demographic and clinical characteristics of patients with first recurrence at the time of initial diagnosis (n=52).

Characteristics Values	
**Age (years)** Mean±SD (min-max)	53.4±12.6 (33-82)
**Tumor size (mm)** Mean±SD (min-max)	99.7±70.3 (15-300)
**Menopause** Yes No	28 (53.8%) 24 (46.2%)
**Symptom** Pain Abdominal distension Postmenopausal bleeding Menorrhagia No symptom Other Not reported	16 (30.8%) 9 (17.3%) 6 (11.5%) 9 (17.3%) 4 (7.7%) 3 (5.8%) 5 (9.6%)
**Hysterectomy** Yes Previously Hysterectomised No	47 (86.5%) 2 (3.9%) 5 (9.6%)
**Lymphadenectomy** Pelvic Pelvic + para-aortic Not performed	4 (7.7%) 33 (63.5%) 15 (28.8%)
**Tumor side** Right Left Bilaterally Not reported	19 (36.6%) 21 (40.4%) 6 (11.5%) 6 (11.5%)
**Capsule rupture (pre/intraoperative)** Yes No Not reported	11 (21.1%) 30 (57.8%) 11 (21.1%)
**Ascites** Yes No Not reported	8 (15.4%) 26 (50.0%) 18 (34.6%)
**Stage (FIGO 2014)** 1A 1B 1C 2 3 4 Not reported	15 (28.8%) 1 (1.9%) 9 (17.3%) 2 (3.8%) 17 (32.7%) 1 (1.9%) 7 (13.6%)
**Lymph node metastasis** Yes No	8 (21.6%) 29 (68.4%)
**Number of excised lymph nodes** Median (IQR) (min-max) Pelvic Para-aortic	26.5 (19.5-33.5) (5-50) 14.0 (8.2-18.2) (4-30)
**Endometrial pathology** Benign Hyperplasia Cancer Not reported	23 (44.2%) 9 (17.3%) 1 (1.9%) 19 (36.6%)
**Appendectomy** Yes No	23 (44.2%) 29 (55.8%)
**Omentectomy** Performed Not performed Not reported	41 (78.8%) 10 (19.3%) 1 (1.9%)
**Omental metastasis** Yes No	10 (24.4%) 31 (75.6%)
**CA-125 levels (U/mL)** (all patients) Median (IQR) (min-max)	13.9 (7-81.5) (3-995)
**CA-125 levels** ≤ 35 U/mL >35 U/mL N/A	21 (40.4%) 9 (17.3%) 22 (42.3%)
**CA-125 levels (U/mL)** (above 35 U/mL group) Median (IQR) (min-max)	114.0 (88-191) (75-995)
**Adjuvant treatment** Chemotherapy Chemotherapy + radiotherapy Not received	27 (51.9%) 2 (3.9%) 23 (44.2%)
**Chemotherapy protocol (n:29)** BEP Platinum + taxane Other	16 (55.2%) 10 (34.5%) 3 (10.3%)

**Abbreviation:** BEP, Bleomycin-Cisplatin-Etoposide; CA-125, Cancer antigen 125; FIGO, International Federation of Gynecology and Obstetrics; N/A, not available; SD, Standard Deviation.

**Table 2:** Clinical characteristics of patients (at first and secondary recurrence)

Characteristics	First Recurrence (n:52)	Secondary Recurrence (n:17)
**Recurrence free survival (month)** Median (IQR) (min-max)	52.5 (25-72.5) (3-240)	-
**CA-125 levels (U/mL)** Median (IQR) (min-max)	9.6 (6-9.6) (1-5172)	-
**CA-125 levels** ≤35 U/mL >35 U/mL N/A	26 (50.0%) 5 (9.6%) 21 (40.4%)	- - -
**Tumor size (mm)** Median (IQR) (min-max)	52.5 (30-82.5) (15-250)	-
**Recurrence pattern** Unifocal Multifocal	23 (44.2%) 29 (55.8%)	10 (58.8%) 7 (41.2%)
**Recurrence location** Pelvic Abdominal (only) Abdominal + pelvic Extraabdominal (only) Extraabdominal + pelvic/abdominal	21 (40.4%) 7 (13.5%) 15 (28.8%) 4 (7.7%) 5 (9.6%)	6 (35.2%) 5 (29.4%) 2 (11.8%) 1 (5.9%) 3 (17.7%)
**Surgery** Performed Not performed	44 (84.6%) 8 (15.4%)	12 (70.6%) 5 (29.4%)
**Postoperative residual disease** Yes No	4 (9.1%) 40 (90.9%)	- -
**Salvage chemotherapy** Received Not received N/A	38 (73.1%) 10 (19.2%) 4 (7.7%)	12 (70.6%) 5 (29.4%)
**Chemotherapy protocol** BEP Platinum + taxane Other	13 (34.2%) 17 (44.7%) 8 (21.1%)	5 (41.7%) 4 (33.3%) 3 (25.0%)
**Disease status** Complete response Partial response Stable disease Progressive disease N/A	26 (50.0%) 3 (5.8%) 1 (1.9%) 10 (19.2%) 12 (23.1%)	8 (47.2%) 2 (11.7%) 2 (11.7%) 5 (29.4%)
**Recurrence ^a^** Yes No Progressive disease or death	17 (32.7%) 25 (48.1%) 10 (19.2%)	5 (29.4%) 7 (41.2%) 5 (29.4%)

^a^ 17 patients have secondary recurrence **Abbreviation**: BEP, Bleomycin-Cisplatin-Etoposide; CA-125, Cancer antigen 125; N/A, Not available; SD, Standard Deviation.

**Table 3:** Factors associated with secondary recurrence in patients with recurrent AGCT

	Secondary Recurrence		p-value
	Positive (n:17)	Negative (n:25)	
**Age ^a^**	55.9 ± 11.7	52.2 ± 12.5	.34
**Tumor size ^a^**	76.7 mm±32.8	94.5 mm±76.2	.47
**Menopause ^a^** Yes No	11 (64.7%) 6 (35.3%)	12 (48.0%) 13 (52.0%)	.28
**Rupture ^a^** Yes No	3 (20.0%) 12 (80.0%)	6 (33.3%) 12 (66.7%)	.45
**Ascites ^a^** Yes No	4 (36.6%) 7 (63.4%)	2 (11.7%) 15 (88.3%)	.17
**Lymphadenectomy ^a^** Yes No	11 (64.7%) 6 (35.3%)	17 (68.0%) 8 (32.0%)	.82
**Number of excised node ^a^**	41.5 ± 14.3	34.8 ± 16.0	.33
**Nodal metastasis ^a^** Yes No	3 (27.3%) 8 (72.7%)	1 (5.9%) 16 (94.1%)	.26
**Omental Metastasis ^a^** Yes No	2 (15.4%) 11 (84.6%)	4 (20.0%) 16 (80.0%)	.99
**CA-125 ^a^** ≤35 U/mL >35 U/mL	8 (66.7%) 4 (33.3%)	12 (85.7%) 2 (14.3%)	.36
**Stage (FIGO 2014)** Stage I Stage II-IV	8 (57.1%) 6 (42.9%)	14 (63.6%) 8 (36.4%)	.69
**Adjuvant chemotherapy ^a^** Received Not received	10 (58.9%) 7 (41.1%)	11 (44.0%) 14 (56.0%)	.34
**Recurrence free survival (month)**	46.3 ± 20.3	64.6 ± 52.1	.12
**Recurrence in ≤60 months ^b^** Yes No	14 (82.4%) 3 (17.6%)	15 (60.0%) 10 (40.0%)	.12
**Pelvic recurrence ^b^** Yes No	13 (76.4%) 4 (23.6%)	19 (76.0%) 6 (24.0%)	.17
**Abdominal recurrence^ b^** Yes No	10 (58.8%) 7 (41.2%)	12 (48.0%) 13 (52.0%)	.66
**Extra-abdominal recurrence^ b^** Yes No	3 (17.6%) 14 (82.4%)	2 (8.0%) 23 (92.0%)	.37
**Recurrence pattern ^b^** Unifocal Multifocal	10 (58.8%) 7 (41.2%)	10 (40.0%) 15 (60.0%)	.23
**CA-125 ^b^** ≤35 U/mL >35 U/mL	6 (60.0%) 4 (40.0%)	16 (100%) 0 (0%)	**.01**
**Surgery ^b, c^** Performed Not performed	16 (94.1%) 1 (5.9%)	22 (88.0%) 3 (12.0%)	.63
**Complete cytoreduction ^b^** No Yes	2 (12.5%) 14 (87.5%)	1 (4.5%) 21 (95.5%)	.56
**Salvage chemotherapy ^b^** Yes No	15 (88.2%) 2 (11.8%)	17 (80.9%) 4 (19.1%)	.67

^a^ at diagnosis; ^b^ at first recurrence; ^c^ 42 patients without progressive disease were analyzed **Abbreviation**: CA-125, Cancer antigen 125; FIGO, International Federation of Gynecology and Obstetrics; RFS, Recurrence free survival.

**Table 4:** Results of Kaplan-Meier analysis of factors potentially associated with OS-FR

	Estimate Mean OS-FR (month)	P-value
**Menopause** Yes No	89.3 139.1	.42
**Rupture** Yes No	172.1 97.9	.63
**Ascites** Yes No	113.5 87.3	.66
**Lymph node metastasis** Yes No	97.7 76.1	.96
**CA-125 ^a^** ≤35 U/mL >35 U/mL	162.5 30.5	**<0.01**
**Stage (FIGO 2014)** Stage 1 Stage ≥2	90.6 126.4	.55
**Adjuvant chemotherapy ^a^** Yes No	114.5 88.4	.46
**Recurrence pattern ^b^** Unifocal Multifocal	125.4 92.3	.89
**PFS** ≤ 60 months > 60 months	134.6 81.6	.97
**CA-125 ^b^** ≤35 U/mL >35 U/mL	93.1 44.6	**.04**
**Pelvic recurrence ^b^** Yes No	81.9 144.8	.26
**Abdominal recurrence ^b^** Yes No	88.7 165.6	.56
**Extra-abdominal recurrence ^b^** Yes No	56.3 131.4	.08
**Residual tumor in surgery ^b^** Yes No	26.3 157.5	**<0.01**
**Salvage treatment ^b,c^** Received Not received	128.3 57.2	**<0.01**
**Salvage treatment ^b,d^** Yes No	155.2 94.0	.67
**Salvage treatment ^b,e^** Yes No	51.2 2.0	**<0.01**

^a^ at diagnosis, ^b^ at first recurrence, ^c^ all patients, ^d^ complete cytoreduction group at first recurrence, ^e^ complete cytoreduction or surgical treatment was not performed group **Abbreviation**: CA-125; Cancer antigen 125, FIGO; International Federation of Gynecology and Obstetrics, PFS; Progression free survival

## Data Availability

The data will be shared upon request.
